# Successful treatment of fulminant myocarditis in an adult in emergency department: A case report

**DOI:** 10.1097/MD.0000000000018292

**Published:** 2019-12-10

**Authors:** Danyu Liu, Jun Xu, Xuezhong Yu

**Affiliations:** Department of Emergency, Peking Union Medical College Hospital, Chinese Academy of Medical Sciences and Peking Union Medical College, Beijing, China.

**Keywords:** adult, emergency service, extracorporeal membrane oxygenation, intra-aortic balloon pumping, myocarditis

## Abstract

**Rationale:**

Fulminant myocarditis (FM) has poor prognosis and the usual treatment is inotropes and symptomatic support. The initiation of extracorporeal membrane oxygenation (ECMO) and intra-aortic balloon pumping (IABP) in the emergency department (ED) is a rare event.

**Patient concerns:**

We report the case of a 45-year-old man with a complaint of 4 days of high fever and dry cough in the emergency department.

**Diagnosis:**

Transthoracic echocardiogram and the medical history showed presumptive diagnosis was fulminant myocarditis with cardiogenic shock.

**Interventions:**

The patient's condition deteriorated drastically and ECMO was initiated immediately after admission. He experienced electrical storm twice during ECMO support and was successfully treated with the combination with IABP.

**Outcomes:**

ECMO and IABP were continued for 11 and 14 days respectively. The patient was discharged on the 81th day after admission, with all his laboratory tests returned to normal.

**Lessons subsections:**

The early initiation of ECMO and IABP in the ED is potentially life-saving for suitable patients with FM. It appears promising but has not yet been routinely implemented in underdeveloped and developing countries.

## 1 Introduction

The severity of myocarditis in adults ranges from relatively mild to fulminant course. Fulminant myocarditis (FM) is a life-threatening and sudden-onset disease, but the patients who survive may exhibit a favorable long-term outcome.^[1]^ In adult patients, FM corresponds to about 10% of cases of myocarditis and has a mortality rate of more than 50%.^[2–4]^ FM are mostly caused by virus, followed by bacteria or noninfectious origin.^[5]^ In some patients, the deterioration of cardiac function can be reversed with the timely institution of mechanical circulatory support, including extracorporeal membrane oxygenation (ECMO), intra-aortic balloon pumping (IABP), or the ventricular-assisted device (VAD), which may substantially improve the prognosis.^[6]^ To our knowledge, almost all of the FM patients, who often first presented to the emergency department (ED), were transferred to department of cardiac surgery, cardiology or critical care medicine to receive mechanical circulatory support. There is no report concerning the use of these therapies to treat FM patients in ED in China. We present a case of FM with multiorgan failure (MOF), which was successfully rescued with percutaneous venoarterial ECMO (VA-ECMO) and IABP in department of emergency intensive care unit (EICU). The patient has provided informed consent for publication of the case.

## 2 Case report

**Figure 1:**
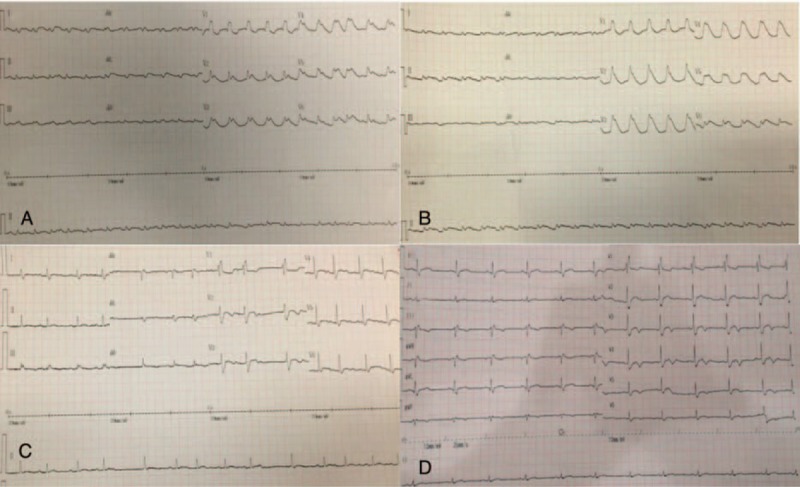
Electrocardiograms during the clinical course. A, ECG at presentation showed sinus tachycardia, complete right bundle branch block, and diffuse ST-segment elevation. B, After the ECMO support, there was wide QRS complex tachycardia. C, After the IABP implantation, there was atrial fibrillation. D, ECG at the time of discharge showed sinus rhythm with complete right bundle branch block. ECMO = extracorporeal membrane oxygenation, IABP = intra-aortic balloon pumping, ECG = electrocardiogram.

The patient was a 45-year-old man, previously healthy, with a complaint of 4 days of high fever and dry cough. When he presented to the emergency room (ER) of our hospital, he was conscious but had severe shortness of breath and associated with nausea and vomiting. He had clammy and cold extremities. Examination showed bilateral wet rales at the bottom of the lungs, regular but fast heart rate, and benign abdomen. Electrocardiogram (ECG) showed sinus tachycardia, complete right bundle branch block, and ST-T changes (Fig. 1A). Chest computed tomography revealed diffuse bilateral patchy infiltrates and ground-glass opacity. Presumptive diagnosis was FM with cardiogenic shock. Continuous oxygen, dobutamine, norepinephrine, diuretic and empiric imipenem and vancomycin, IVIG (20 g per day for 5 days), vitamin C, coenzyme Q 10 were started immediately and we did not use any steroids or immunosuppressive agents. Despite clinical treatment, the patient did not show any improvements. After explaining the disease condition and prognosis to the patient's family, he was transferred to the EICU from the ER in 12 hours. The patient's condition deteriorated drastically and tracheal intubation, VA-ECMO, and continuous renal replacement therapy (CRRT) were initiated 6 hours after EICU admission. A transthoracic echocardiogram (TTE) showed global hypokinesia and severely impaired left ventricular (LV) function with an ejection fraction (EF) of 18%. Tests to identify the etiologic agent were performed and all negative, including enterovirus, parvovirus B19, adenovirus, influenza virus assessed by PCR at nasopharyngeal swab and CMV, EBV, HSV, mycoplasma assessed by serology in the plasma. Laboratory measurements showed thrombocytopenia, elevated levels of cardiac enzyme, liver enzyme, blood urea nitrogen, creatinine, and inflammatory markers (Table 1).

**Table 1:**
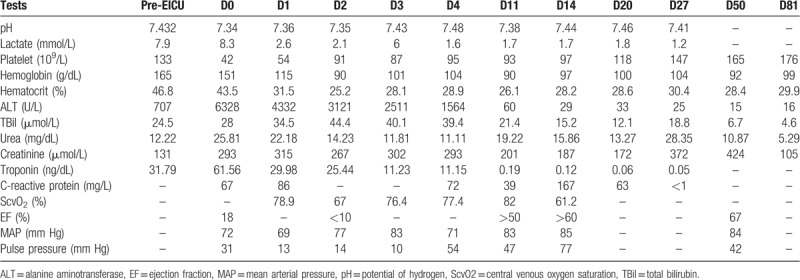
Test results throughout the clinical course.

Heparin was continuously administrated by an infusion pump to maintain an activated partial thromboplastin time (APTT) of 70 to 100 seconds. To guarantee the level of hemoglobin > 9 g/dL and platelet > 50,000/μL, blood transfusion was administered intermittently. To prevent the distal leg ischemia, a catheter connected superficial femoral artery with femoral artery was required. To recover from peripheral circulatory failure, the initial flow rate was set as 4.5 L/min. His cardiac enzyme and liver enzyme gradually declined to normal.

**Figure 2:**
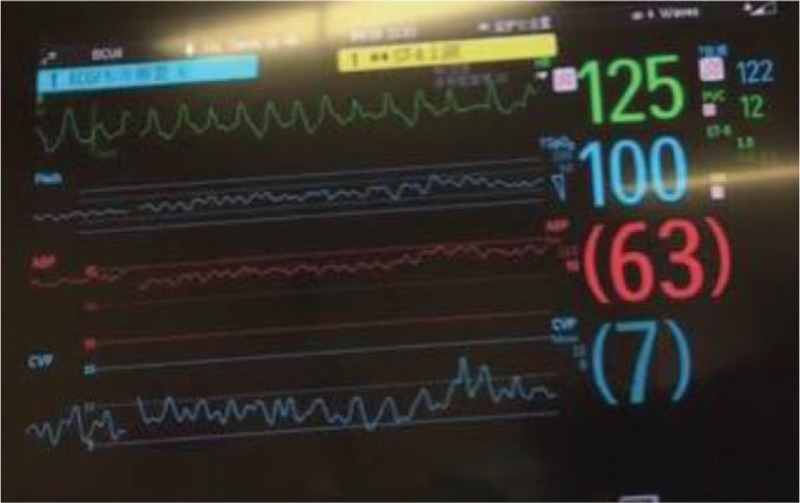
Electrical storm after ECMO. ECMO = extracorporeal membrane oxygenation.

**Figure 3:**
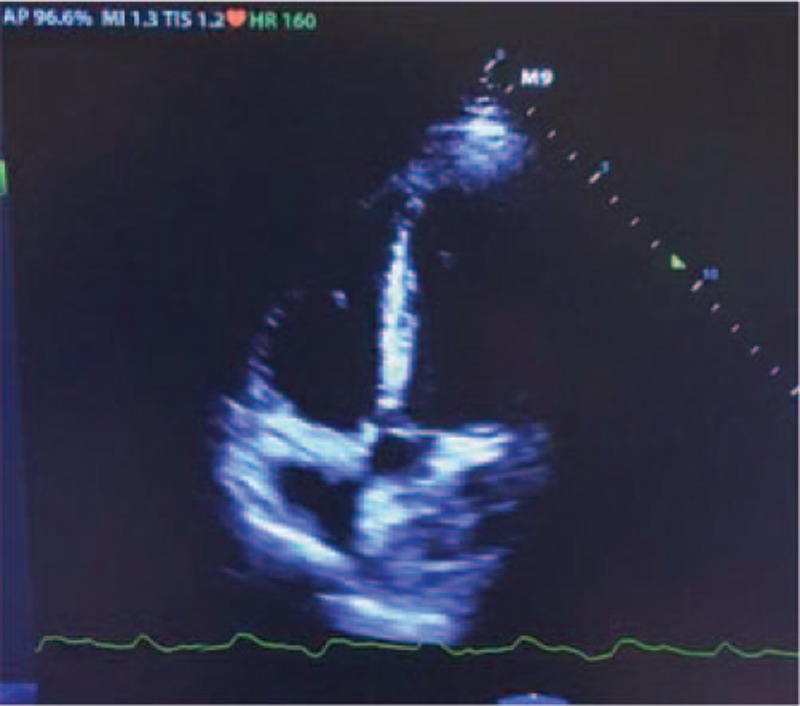
Echocardiography showed aortic valve hardly opened during left ventricular contraction.

On the first day of EICU admission and after ECMO administration, the patient experienced short ventricular tachycardia (VT) which could be reversed by antiarrhythmic drugs (Fig. 1B). However, he experienced refractory VT and ventricular fibrillation (VF) 2 days later. Unfortunately, the electrical storm could not be reversed by the electric cardioversion, defibrillation, cardiopulmonary resuscitation (CPR), and antiarrhythmic drugs (Fig. 2). The bedside echocardiography showed LVEF was lower than 10% and aortic valve hardly opened during left ventricular contraction, so IABP was administered with an initial pace ratio of 1:2 on the following day (Fig. 3). After the IABP support, he experienced atrial fibrillation which could be reversed by antiarrhythmic drugs (Fig. 1C).

Daily bedside echocardiography demonstrated gradual improvement in cardiac contractility. The flow rate of ECMO and the pace ratio of IABP was decreased, according to the vital signs and indicators of peripheral circulatory failure (e.g., lactic acid, mixed venous oxygen saturation, and arterial blood gas analysis).

ECMO was able to be withdrawn on hospital day 11 when transesophageal echocardiogram (TEE) showed estimated LVEF of 50% and the patient was weaned from IABP successfully on hospital day 14. He was extubated on hospital day 20 but required tracheotomy. The patient stayed in EICU for 33 days and was discharged on the 81th day after admission, with all his laboratory tests returned to normal (Table 1). Electrocardiogram showed sinus rhythm with complete right bundle branch block (Fig. 1D). Echocardiography showed normal cardiac chambers and normal wall motion with EF 67%. His stay in the hospital was complicated by hospital-acquired pneumonia, acute renal failure, gastrointestinal bleeding, and critical illness myopathy.

## 3 Discussion

The clinical characteristics of FM are not specific. Patients often present with nonspecific flu-like symptoms initially, but rapidly progress to sudden onset of cardiac symptoms, such as severe heart failure, cardiogenic shock, and potentially fatal arrhythmias, which can lead to significant mortality.^[5]^ Histology is relevant in the assessment of fulminant myocarditis as giant cell myocarditis have generally a poorer prognosis compared with eosinophilic and lymphocytic myocarditis. However, it was a pity that biopsy was not performed in this case because of hemodynamic instability and anticoagulation. The blood analysis of virus is not sufficient and advances in molecular detection of viruses by endomyocardial biopsy may be helpful.^[7]^

In this case we did not use any immunosuppressive treatment, such as steroids and azathioprine, because it was still controversial.^[8,9]^ Although we used IVIG to regulate the immune system, further investigations are warranted to demonstrate whether there are any potential benefits. Mechanical circulatory support including ECMO, IABP, or the VAD can be lifesaving. VA-ECMO is an effective approach for reestablishing cardiac output and organ perfusion in patients with FM. An overall survival rate of 59% to 71% was seen in adults with FM supported with VA-ECMO.^[1,10–12]^ However, the starting time of VA-ECMO is still controversial. VA-ECMO should be considered if the circulation is still inadequate after conservative therapy.^[13]^ Bedside echocardiography should be undertaken daily to see whether the ventricular systolic function is recovered. Once there is durable evidence of cardiac recovery, trails of discontinuation may be undertaken. One of the characteristics of this case is that the duration of VA-ECMO assistance is 11 days, which is a little longer than that reported in previous cases (6–9 d).^[12]^ Maybe this is related to the serious condition of the patient. At the same time, it can help doctors build experience on the long-term VA-ECMO support of FM (11 d).

VA-ECMO can lead to LV overload and distention, whereas concomitant IABP can reduce LV afterload and increase coronary blood flow. In addition, the increase in LV end-diastolic pressure can reduce coronary blood flow and cause electrical storm.^[14]^ Thus, maybe this is the reason the patient experienced the electrical storm twice in this case and it may reinforce the importance of early combined use of VA-ECMO and IABP, which is consistent with the findings of Hu et al.^[15]^

Most studies found that the survival rate to hospital discharge of patients with FM was higher than refractory postcardiotomy cardiac failure and acute myocardial infarction.^[1,16–18]^ As a temporary and transitional method, successful weaning of ECMO depends on whether the cause of heart failure can be treated timely or the cardiac function can be improved on its own. Therefore, before treatment with ECMO, it is necessary to consider whether the cardiac function of patients is reversible, whether the cause can be treated, and whether further treatment can be followed. In this case, the patient had a good prognosis, mainly because myocardial inflammation is self-limited and recoverable. However, not all FM patients can survive with the treatment of mechanical circulatory support and have a favorable prognosis. Ammirati et al^[19]^ found patients with FM had a significantly increased mortality and need for heart transplantation compared with those with non-FM. Lorusso et al^[1]^ demonstrated that lactate normalization hours from ECMO implantation, pH before ECMO implantation, and cardiac function recovery were independently related to prognosis. Saito et al^[20]^ showed that the duration of ECMO support was significantly associated with the total bilirubin level. FM patients, who have initiated several days of support by ECMO without improvement in cardiac function, should change to VAD therapy.^[21]^ Therefore, selecting the suitable patients before the use of ECMO may be important to increase the survival rate.

## 4 Conclusion

FM is a grievous condition with high mortality. No delay should be had on starting VA-ECMO if suspected in suitable patients. Early combined use of IABP may reduce the incidence of electrical storm during ECMO support. This is our first experience of using ECMO and IABP in the ED. The reported patient survived with all his laboratory tests returned to normal. The use of mechanical circulatory support appears promising but has not yet been routinely implemented in underdeveloped and developing countries.

## Author contributions

**Conceptualization:** Danyu Liu, Jun Xu, Xuezhong Yu.

**Data curation:** Danyu Liu, Jun Xu.

**Formal analysis:** Danyu Liu, Jun Xu, Xuezhong Yu.

**Funding acquisition:** Danyu Liu, Jun Xu, Xuezhong Yu.

**Investigation:** Danyu Liu, Jun Xu, Xuezhong Yu.

**Methodology:** Danyu Liu, Jun Xu, Xuezhong Yu.

**Project administration:** Danyu Liu, Jun Xu, Xuezhong Yu.

**Resources:** Danyu Liu, Jun Xu, Xuezhong Yu.

**Software:** Danyu Liu, Jun Xu, Xuezhong Yu.

**Supervision:** Danyu Liu, Jun Xu, Xuezhong Yu.

**Validation:** Danyu Liu, Jun Xu, Xuezhong Yu.

**Visualization:** Danyu Liu, Jun Xu, Xuezhong Yu.

**Writing – original draft:** Danyu Liu.

**Writing – review & editing:** Danyu Liu, Jun Xu, Xuezhong Yu.
